# Draft Genome Sequences of Three *Chromobacterium subtsugae* Isolates from Wild and Cultivated Cranberry Bogs in Southeastern Massachusetts

**DOI:** 10.1128/genomeA.00998-15

**Published:** 2015-09-10

**Authors:** Kristin Vöing, Alisha Harrison, Scott D. Soby

**Affiliations:** a Fachbereich Biologie, Universität Münster, Münster, Germany; b Biomedical Sciences Program, College of Health Sciences and College of Veterinary Medicine, Midwestern University, Glendale, Arizona, USA

## Abstract

*Chromobacterium subtsugae* was isolated from cranberry bogs in Massachusetts. While it is unknown what environmental role these bacteria play in bog soils, they hold potential as biological control agents against the larvae of insect pests. Potential virulence genes were identified, including the violacein synthesis pathway, siderophores, and several chitinases.

## GENOME ANNOUNCEMENT

*Chromobacterium subtsugae* strains were isolated from cultivated (MWU3525) and wild (MWU2576, MWU2920) cranberry bog soils in Massachusetts, and tentatively identified as *C. subtsugae* by phenotype and by 16S rRNA sequences (1–3). Their genomes were sequenced at the University of Arizona Genetics Core using a 454 GS FLX Titanium following manufacturer protocols. Libraries were generated using the NEBNext Quick DNA Library Prep Master Mix set for 454, and MID tagged using the Library Prep Kit Rapid Library MID adapters kit. The emPCR Amplification Method manual, Lib-L LV, XL+ (June 2013), was followed for bulk emPCR of each sample. Three samples were loaded into each region of a sequencing PTP plate divided with a 2-region gasket. Roughly 670,000 beads were loaded per sample. Roche Newbler Software v2.9 was used for signal processing, sample demultiplexing, and partial assembly. Assembly data for all three isolates are given in Table 1. Isolate genomes were compared to each other and to reference genomes of *C. violaceum* (ATCC 12472), *C. haemolyticum* (T124), *C. vaccinii* (MWU205), and *C. subtsugae* (F49) using the Genome-to-Genome Distance Calculator (GGDC) provided online by the DSMZ. GGDC mimics *in vitro* DNA-DNA hybridization by dividing scaffold sequences into fragments approximately the same size as would be expected *in vitro*, and pairing up homologous segments (4–6). MWU2576, MWU2920, and MWU3525 total genomes were 87.6 to 95.3% homologous by this method, 90 to 96% homologous to the *C. subtsugae* reference genome, confirming them as a member of this species. In contrast, they were 30% homologous to the type species/isolate of the genus, *C. violaceum* ATCC 12472.

**TABLE 1:** Accession numbers and genome sequencing statistics

Isolate	Accession no.	No. of reads per sample	Coverage	No. of scaffolds/large scaffolds	No. of nucleotides (nt) on large scaffolds	*N*_50_	Maximum scaffold length (nt)	G+C fraction (%)	Estimated genome size (MB)
MWU2576	LCWQ00000000	245,078	618.6×	82/61	4,803,580	175,428	394,904	64.76	5.4
MWU2920	LCWP00000000	243,888	625.8×	86/50	4,665,268	222,142	339,870	64.86	5.1
MWU3525	LCWO00000000	224,323	609.2×	103/63	4,700,397	181,598	397,271	64.83	5.5

*Ab initio* gene prediction was performed on the assembly using RAST (http://rast.nmpdr.org/). A number of potential virulence factor genes were found that may contribute to larval toxicity, including production of the pigment violacein, siderophores, hydrogen cyanide, and secreted chitinases (7). MWU2576 contained 16 probable chitinase genes, including only one probable chitinase A gene, and six endochitinases. MWU2920 contained 14 probable chitinase genes, including three probable chitinase A genes, and two endochitinases. This isolate was unusual in that it also contained ten genes for chitinase family 18 proteins. MWU3525 contained 17 probable chitinase genes, including nine probable chitinase A genes, and seven endochitinases. In contrast to *C. vaccinii* (8, 9), none of the isolates contained putative hydrolase transmembrane family proteins.

### Nucleotide sequence accession numbers.

This whole-genome shotgun project has been deposited at DDBJ/EMBL/GenBank under the accession numbers listed in Table 1. The versions described in this paper are versions LCWQ01000000, LCWP01000000, and LCWO01000000.
